# Dopaminergic neurons regenerate following chemogenetic ablation in the olfactory bulb of adult Zebrafish (*Danio rerio*)

**DOI:** 10.1038/s41598-020-69734-0

**Published:** 2020-07-30

**Authors:** Rafael Godoy, Khang Hua, Michael Kalyn, Victoria-Marie Cusson, Hymie Anisman, Marc Ekker

**Affiliations:** 1 Department of Biology, University of Ottawa, Ottawa, ON Canada; 2 Department of Neuroscience, Carleton University, Ottawa, ON Canada

**Keywords:** Neuroscience, Regeneration and repair in the nervous system

## Abstract

Adult zebrafish have the ability to regenerate cells of the central nervous system. However, few neuronal regeneration studies in adult zebrafish addressed their ability to regenerate specific types of neurons following cell specific ablation. We show here that treatment of transgenic Tg(*dat:CFP-NTR*) adult zebrafish with the prodrug metronidazole (Mtz) according to our administration regimen predominantly ablates dopamine (DA) neurons within the olfactory bulb (OB) of adult fish. Loss of DA neurons was accompanied by an impaired olfaction phenotype, as early as 1-week post-treatment, in which fish were unable to sense the presence of the repulsive stimulus cadaverine. The olfactory impairment was reversed within 45 days and coincided with the recovery of DA neuron counts in the OB. A multi-label pulse-chase analysis with BrdU and EdU over the first seventeen days-post Mtz exposure showed that newly formed DA neurons were recruited within the first nine days following exposure and led to functional and morphological recovery of the OB.

## Introduction

Over the last decade, the zebrafish has emerged as a strong model for the study of neurogenesis and brain regeneration as previously reviewed^1–3^. Unlike mammals, which have two main proliferation zones—the subgranular and subventricular zones^4,5^, the zebrafish holds 16 different zones of proliferation in the brain^6,7^. This ability to hold numerous cells in a proliferating stage allows for the recruitment of stem-progenitor cells to multiply and migrate following damage to brain tissue in order to allow cells to then remodel the brain environment in a regenerative fashion^8,9^. While much of our knowledge about the regenerative capacity of the adult zebrafish brain have come from invasive stab lesion studies^8–11^, few studies have investigated the zebrafish ability to regenerate a specific neuronal subtype through a conditional and specific ablation approach in the adult brain.

Neurotoxins such as MPTP (1-methyl-4-phenyl-1,2,3,6-tetrahydropyridine) and 6-OHDA (oxidopamine) have been classically used for the ablation of DA neurons in animal models of Parkinson’s disease (PD)^12–15^. Some of these drug treatments failed to produce changes in TH-positive neuronal population arrangement^16,17^. Others showed variable ability of DA neurons to regenerate^15^.

Dopaminergic neurons have been described in the olfactory bulb, pretectum, telencephalon, preoptic area and diencephalon of the adult zebrafish brain. Whereas some clusters of dopaminergic neurons of the teleost diencephalon are thought to regulate movement similarly to the mammalian striatum; DA neurons of the OB carry sensory response to odor. Overall, brain DA is involved in the regulation of movement, emotion, reward, memory, attention, motivation and hormonal secretion.

Chemogenetic ablation has emerged as an alternative to the ablation of DA neurons with neurotoxins, which are toxic to the animals and provided variable results in zebrafish. The chemogenetic ablation method based on expression of nitroreductase (NTR) allows for the conditional and specific induction of cell death mediated via the caspase-activated cascade^18,19^. The technique relies on the enzyme NTR, expressed under the control of regulatory elements from genes expressed in a tissue-specific manner. We and others have used NTR to successfully ablate DA neurons in juvenile zebrafish^20–24^. There has been evidence for the recovery of lost cells in juvenile zebrafish following chemogenetic ablation but so far, the ability of adult zebrafish to regenerate DA neurons had not been demonstrated. Here, we use the Tg(*dat:CFP-NTR*) transgenic line that we previously used to ablate DA neurons of juveniles^22^ and show that administration of the NTR substrate metronidazole (Mtz) to adult zebrafish leads to a large depletion of DA neurons in the olfactory bulb. This neuronal loss is accompanied by decreases in dopamine neurotransmitter content and impaired olfactory behavior. Both cell counts and phenotype are recovered after removal of Mtz, indicative of regeneration of DA neurons.

Pulse chase lineage tracing experiments showed that recruitment of progenitor cells occurs within the first nine days post exposure, showing that upon injury there is an immediate recruitment of cells with the ability to give rise to functional DA neurons in the adult zebrafish OB.

## Results

### Ablation of DA neurons in the olfactory bulb of adult Tg(*dat:cfp-ntr*) zebrafish

**Figure 1:**
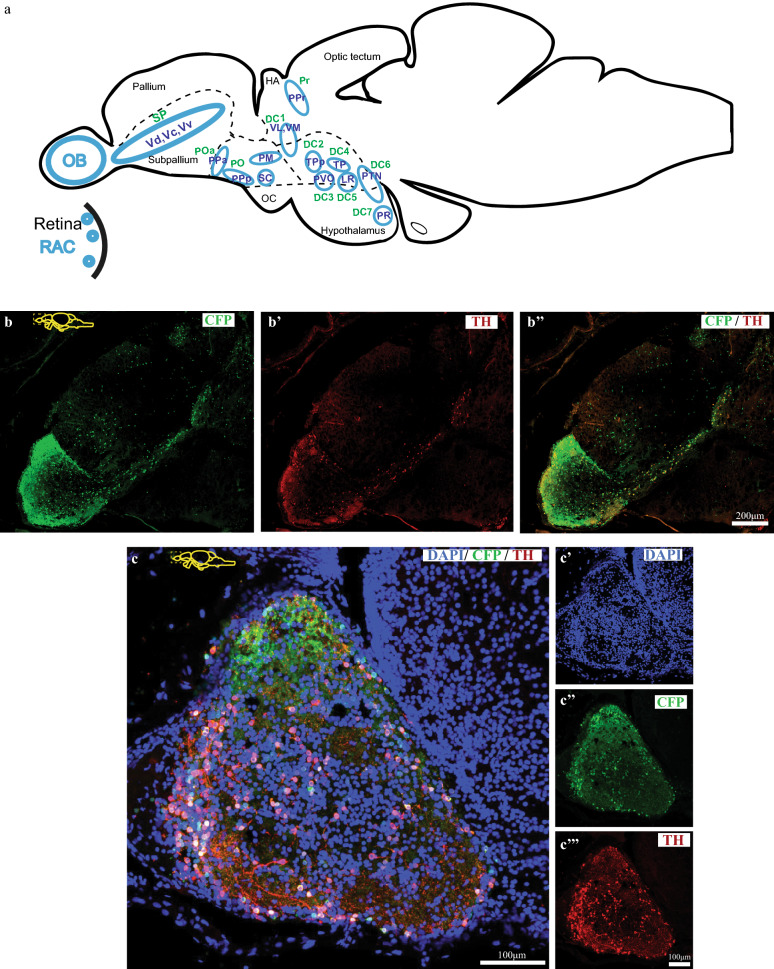
Expression of Tg(dat:CFP-NTR) in the adult zebrafish brain. (**a**) Schematic representation of DA clusters expressing CFP-NTR. These include the amacrine cells of the retina (RAC), cells of the olfactory bulb (OB), of the ventral telencephalon (Vd, Vc and Vv), of the preoptic area (POa, PO), periventricular pretectal nucleus (PPr) and DA-producing cells of the diencephalon (DC). (**b**) Sagittal section of the telencephalon showing CFP-expressing cells (green) and co-expression with TH (red). (**c**) Sagittal section of adult zebrafish olfactory bulb showing cell nuclei (DAPI) (**c**′), CFP (**c**″) and TH (**c**‴). (**d**) Transverse section of a medial olfactory bulb section showing expression of DAPI (**d**), CFP (green) (**d**′) or TH (red) (**d**″) and its colocalization (**d**‴); followed by its magnified image (**e**–**e**‴).

**Figure 2:**
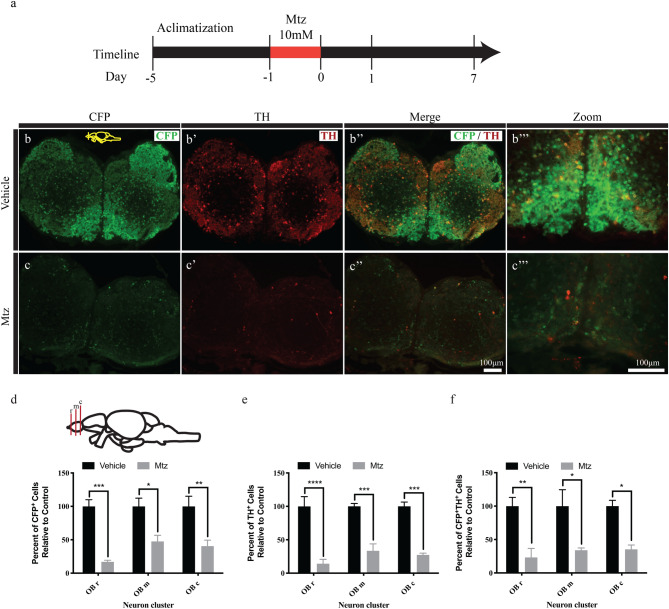
Ablation of DA neurons following Mtz treatment. (**a**) Timeline of experiment. (**b**–**b**‴) Representative immunofluorescence of CFP positive (Green) and TH positive (Red) cells in the OB of vehicle control fish. (**b**–**b**‴). Ablation of CFP (Green) and TH (Red) positive cells in the OB of Mtz-treated fish (**c**–**c**‴). (**d**–**f**) Quantification of the number of CFP^+^ (**d**), TH^+^ (**e**), and CFP + /TH + cells expressing CFP and TH (**f**). Quantification was conducted in 3 areas of OB, a rostral (OBr), a medial (OBm) and a more caudal (OBc) area. *(*p* ≤  0.05), **(*p* ≤  0.01), ***(*p* ≤  0.001) and ****(*p* ≤  0.0001).

Expression of the transgene, *cfp-ntr*, was previously reported in dopaminergic neurons of embryonic and larval transgenic, Tg(*dat:CFP-NTR*) zebrafish^22^. As depicted in Fig. 1, expression of the transgene persists in the adult zebrafish brain and, similarly to larval expression, has been detected in the olfactory bulb (OB), in the ventral telencephalon and in the diencephalon (Fig. 1a). Sagittal view of adult forebrain shows localization of CFP across both OB and telencephalon (Tel) (Fig. 1b) with CFP expression co-localizing with another DA neuron marker, tyrosine hydroxylase (TH) (Fig. 1b′ and b″). Although most TH immunoreactive cells express CFP (Fig. 1c), the expression of only CFP in some cells can be explained by the presence of two TH paralogs in zebrafish (*th1* and *th2*)^25^ and by the fact that our TH antibody only recognizes one of the two paralogs. Cross sectional view of the OB (Fig. 2b) further shows CFP/TH expression profiles in the adult zebrafish brain.

We next tested if treatment of adult Tg(*dat*:*CFP-NTR*) zebrafish with Mtz resulted in DA neuron ablation, as previously observed in juvenile zebrafish^22^. Administration of Mtz (10 mM) for 24 h (Fig. 2a) was sufficient to cause ablation of DA neurons in the adult OB when measured 7 days following exposure (Fig. 2b,c). Mtz treatment decreased both CFP and TH immunoreactivity (Fig. 2b,c) in the adult OB. Quantification of the degree of neuron ablation following Mtz treatment resulted in 83, 53 and 60% reductions in the number of CFP^+^ cells in the rostral, medial and caudal portion of OB respectively (n = 3, *p* = 0.0003, *p* = 0.0117 and *p* = 0.005 respectively) when compared to vehicle treated animals (Fig. 2d). The number of TH-labeled cells was also reduced across the Olfactory Bulb, rostral to caudal (OBr-c) by 86, 67 and 73% (n = 3, *p* = 0.0001, *p* = 0.0004 and *p* = 0.0002 respectively) in Mtz-treated animals (Fig. 2e). Furthermore, a similar trend was observed when looking at double labeled, CFP and TH positive cells, where there was a 76.8, 66 and 65% reduction (n = 3, *p* = 0.005, *p* = 0.014 and *p* = 0.016 respectively) in the number of CFP^+^/TH^+^ cells (Fig. 2f).

Loss of DA neurons was observed in other regions of the adult zebrafish brain, mainly in the telencephalon (data not shown) but not to the same extent as in the olfactory bulb. The reasons for these differences are presently unclear but may be related to the method of Mtz administration in the fish water resulting in insufficient penetration of the drug throughout the whole brain.

**Figure 3:**
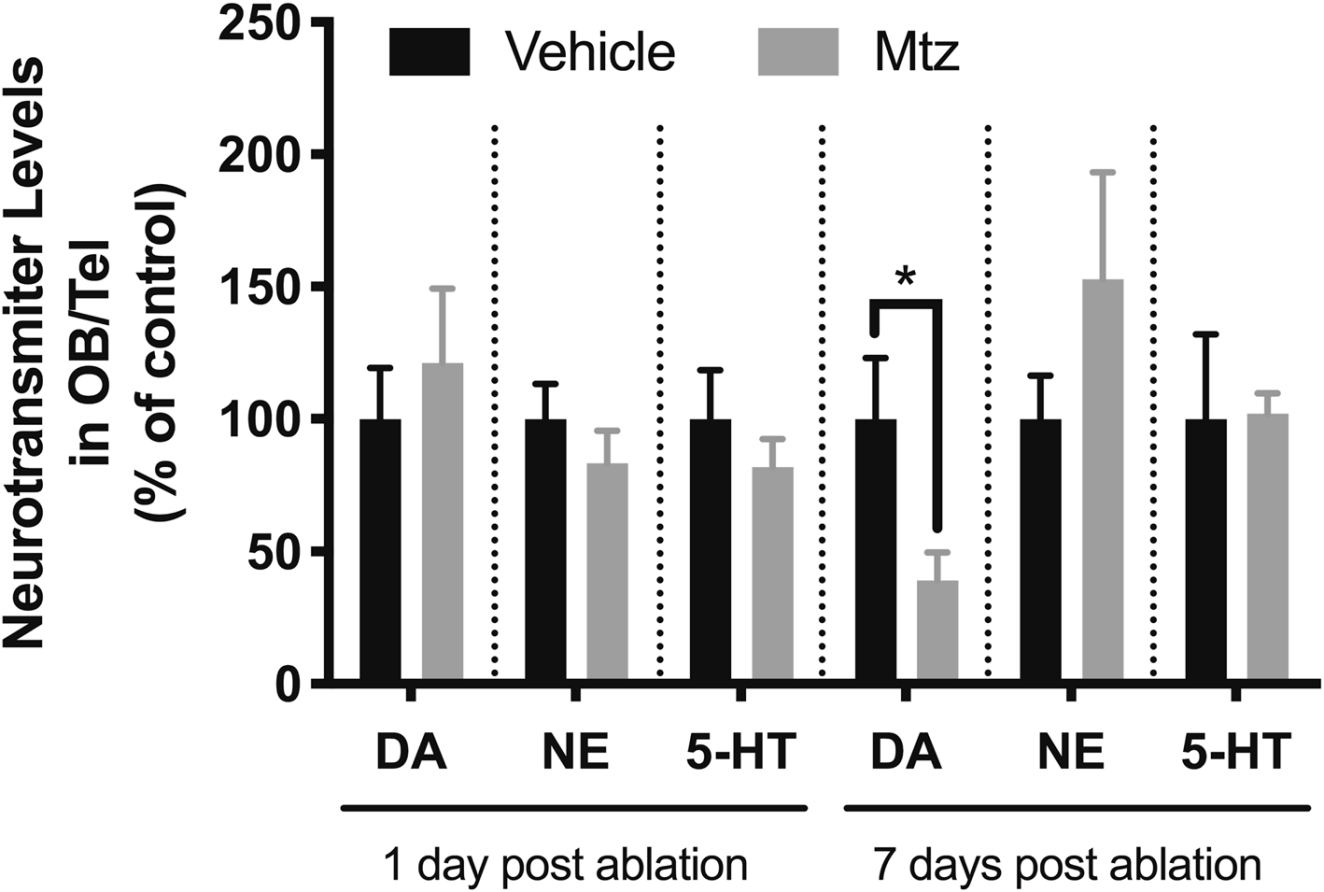
Decrease in neurotransmitter levels following ablation of dopaminergic neurons. Levels of dopamine (DA), serotonin (5-HT) and norepinephrine (NE) were measured in dissected OB/telencephalon tissue, 1 or 7 days post-Mtz treatment. *(*p* ≤  0.05).

To determine whether the loss of CFP^+^ neurons also resulted in a decrease in dopamine levels, we measured the levels of dopamine within the OB/telencephalon area by HPLC analysis of dissected brain tissue. Seven days following the end of Mtz treatment, there was a 61% decrease in dopamine levels in the OB/Tel area (n = 6, *p* = 0.04) (Fig. 3). The levels of other neurotransmitters such as norepinephrine (NE) and serotonin (5-HT) remained unaffected. This suggests that Mtz-mediated ablation in Tg(*dat*:*CFP-NTR*) zebrafish affects DA neurons predominantly, if not exclusively.

### Impaired olfactory behaviour following NTR-Mtz-mediated ablation of DA neurons

**Figure 4:**
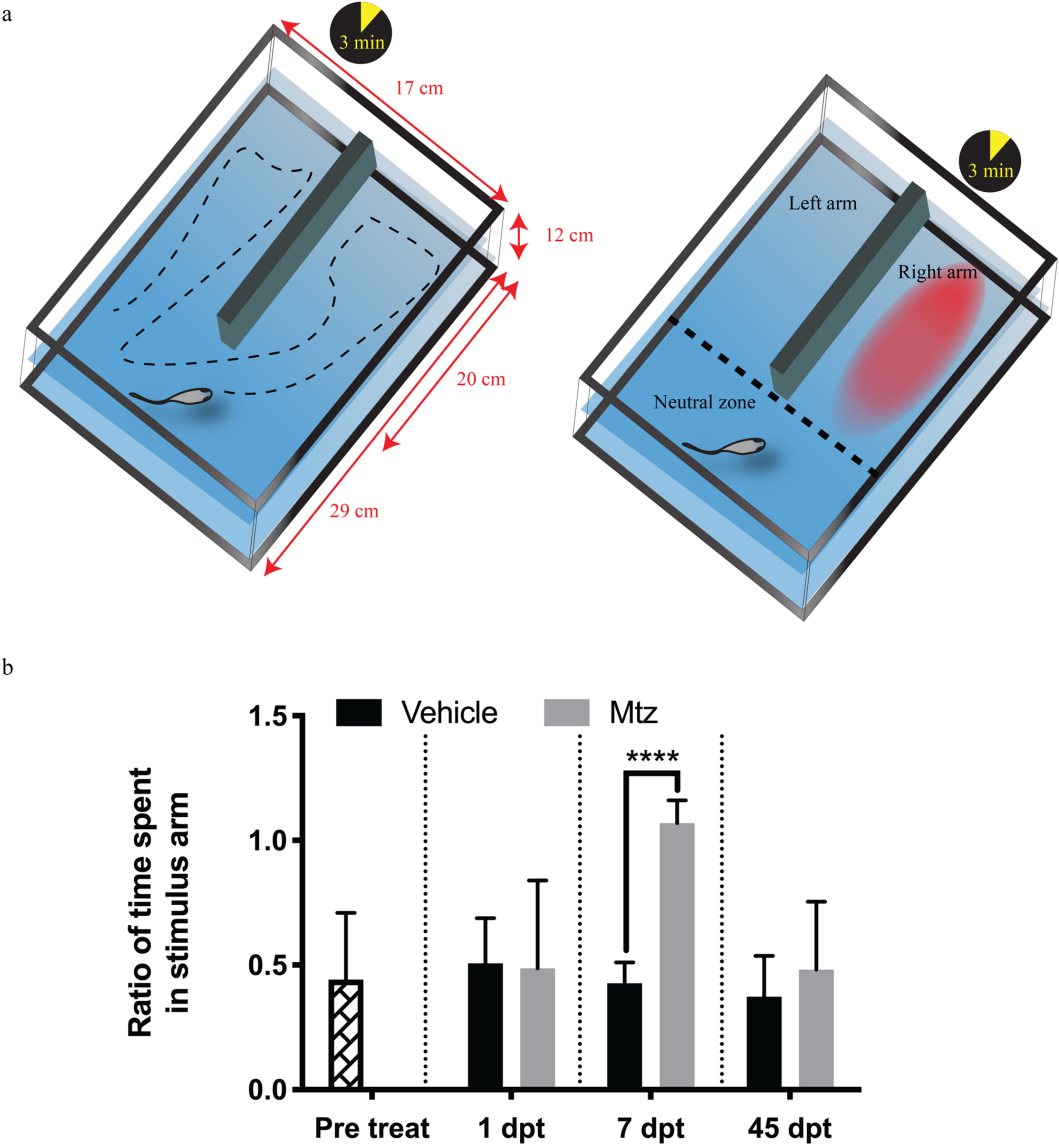
Decreased olfactory function following neuronal ablation. (**a**) Schematic representation of the tank designed to address OB phenotype. The tank contained a mid-tank wall separating space into 3 areas: left arm, right arm and neutral zone. Animals were allowed to swim freely for 3 min prior to the addition of repulsive stimulus cadaverine. Animal response to the stimulus was recorded for 3 additional minutes. (**b**) Animals tend to spend equal time swimming across different zones of tank prior to stimuli. Upon stimulus, control fish decrease total time spent in the stimulus arm, whereas this ability is impaired upon Mtz treatment at 7 days post ablation but recovered by day 45. ****(*p* ≤  0.0001). (Sample size of n = 6 animals for each treatment and time poit except for 1 dpt where n = 3, statistical power was calculated using a multiple *t *test corrected for multiple comparison using Holm–Sidak post hoc).

To determine if the loss of DA neurons in the OB of adult Tg(*dat*:*CFP-NTR*) zebrafish has functional implications, we carried out a repulsive stimulus test. Adult fish were placed in a two-arm tank (Fig. 4a). Following acclimatization, a repulsive stimulus (cadaverine), which has been previously shown to induce a repulsive behaviour in zebrafish^26^, was added to the arm in which the fish was located. We then recorded the time fish spent in each section of the tank and calculated the ratio of time spent in the stimulus arm. Prior to neuronal ablation, fish tend to spend less time in the stimulus arm in response to the repulsive scent of cadaverine (n = 6, mean ratio = 0.45) (Fig. 4b). This repulsive phenomenon was still observed shortly after Mtz administration (1 dpt) (n = 3, Vehicle-mean ratio = 0.50, Mtz-mean = 0.49) but correlating with the peak in neuronal ablation at day 7, Mtz-treated animals lost their ability to sense the repulsive scent of cadaverine as indicated by the longer time spent in the stimulus arm (n = 3, Vehicle-mean ratio = 0.43, Mtz-mean ratio = 1.07, *p* = 0.0004) (Fig. 4b). This suggests that neuronal ablation resulted in the inability of fish to either detect cadaverine or relay the information to the rest of the brain. The impairment of the olfactory system was transient as the fish had mostly recovered and were able to generate a normal response to the repulsive stimulus at 45 dpt (Fig. 4b).

**Figure 5:**
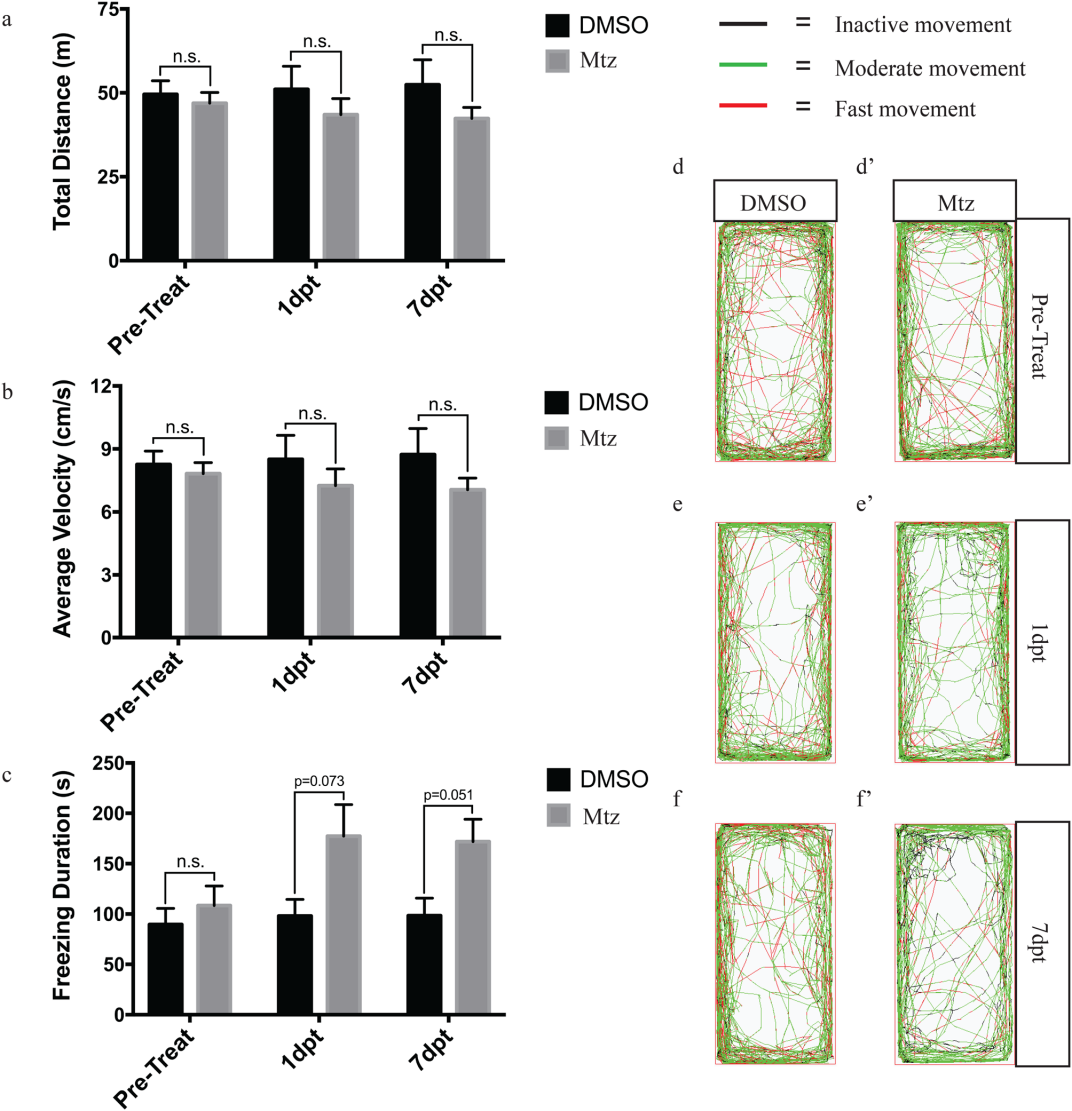
Relative swimming activity of Mtz and DMSO treated adult zebrafish**.** All zebrafish were transferred to individual tanks to acclimate for 30 min prior to behavioural analyses. (**a**–**c**) Effects of Mtz and DMSO on total distance travelled, average velocity, and freezing bout duration. (**d**/**d**′–**f**/**f**′) Overhead path images of adult zebrafish assessed at 3 time points; pre-treatment, 1 dpt, and 7 dpt. Inactive movements (0–4 cm/s), moderate movement (4–8 cm/s) and fast movement (> 8 cm/s). The graphs were made with GraphPad Prism 7.0 and the path images were generated with the ZebraLab software and ZebraCube tracking system (ViewPoint Life Science, Lyon, France). Sample sizes of n = 10; bars represent the Mean ± the SEM; statistical power calculated using a two-way ANOVA followed by Tukey’s multiple comparison test.

We also analyzed the swimming/locomotor behavior of Tg(*dat*:*CFP-NTR*) fish from 1 day post treatment until 7 dpt., including total distance swam, average velocity and freezing duration (Fig. 5),. There was no observable locomotor phenotype in Mtz-treated animals except for some increase in freezing duration (Fig. 5c).

### Recovery in olfactory sensitivity coincides with dopaminergic cell regeneration

**Figure 6:**
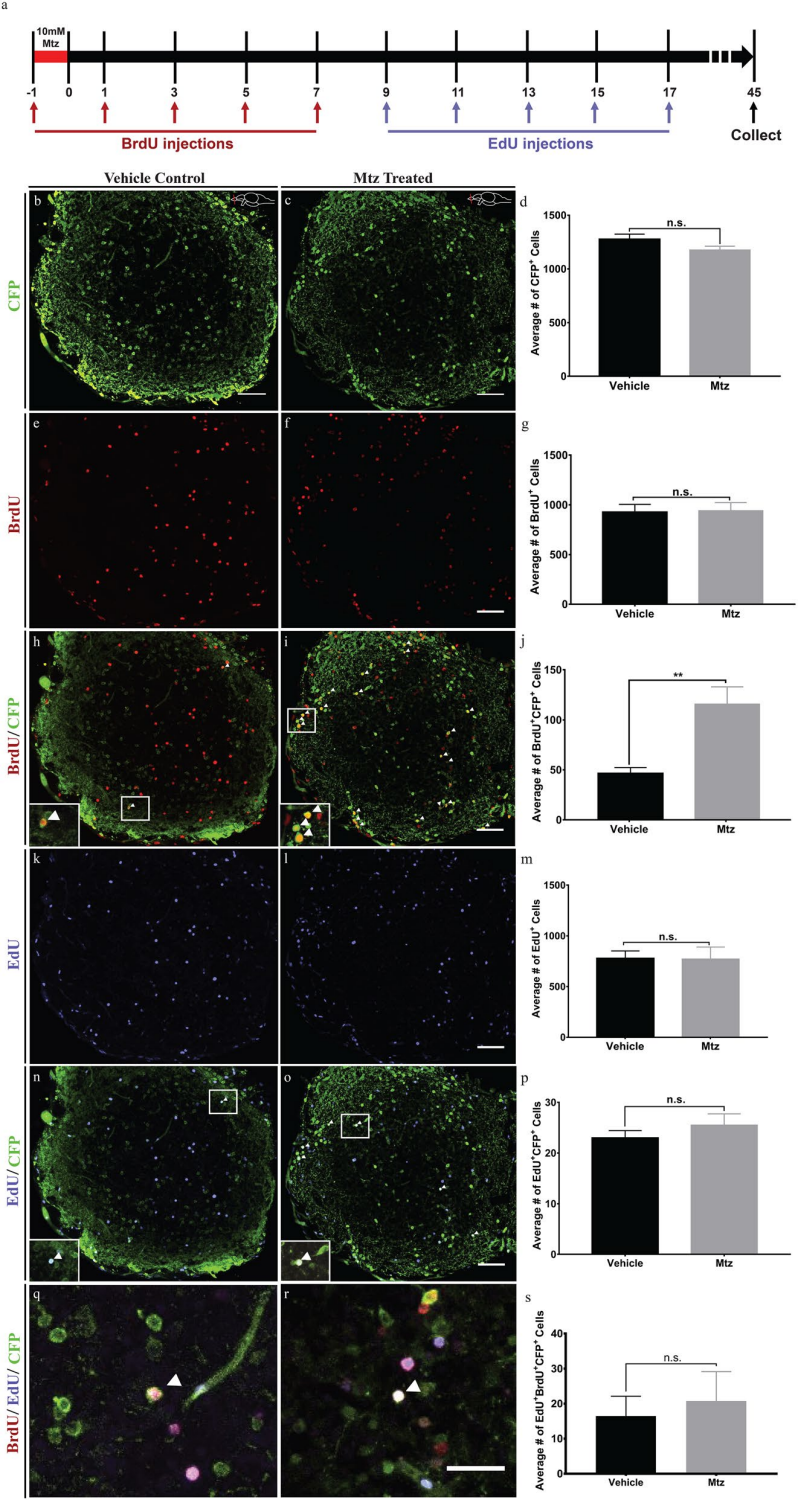
Formation of new CFP positive cells following neuronal ablation only occur before 9 days post ablation. (**a**) Timeline of experiment. (**b**, **c**) Representative images of a coronal OB section from Vehicle control (**b**) and Mtz treated (**c**) fish with cells expressing CFP. (**d**) Quantification of the number of CFP positive cells showing no significant difference between Mtz treated and control fish (n = 6). (**e**, **f**) Representative images of a coronal OB section from Vehicle control (**e**) and Mtz treated (**f**) fish with cells positive for BrdU. (**g**) Quantification of the number of BrdU positive cells relative to control. There was no significant difference between Mtz treated fish relative to control (n = 6). (**h**, **i**) Representative images of a coronal OB section from Vehicle control (**h**) and Mtz treated (**i**) fish with cells expressing. (**j**) Quantification of the number of cells positive for both BrdU and CFP relative to control. There was a 2.5-fold increase in the number of double labelled cells in Mtz-treated fish relative to control (n = 6). (**k**, **l**) Representative images of a coronal OB section from Vehicle control (**k**) and Mtz treated (**l**) fish with cells positive for EdU. (**m**) Quantification of the number of EdU positive cells relative to control. There was no significant difference between Mtz treated and control fish (n = 6). (**n**, **o**) Representative images of a coronal OB section from Vehicle control (**n**) and Mtz treated (**o**) fish with cells positive for both EdU and CFP. (**p**) Quantification of the number of EdU and CFP positive cells relative to control. There was no significant difference between Mtz treated and control fish (n = 6). (**q**, **r**) Representative images of a coronal OB section from Vehicle control (**q**) and Mtz treated (**r**) fish positive for BrdU, EdU, and CFP. (**s**) Quantification of the number of triple-labelled cells relative to control. There was no significant difference between Mtz treated fish relative to control. **P < 0.01, n.s. = not significant, N = 6. Scale bar: 50 μm.

Mtz-treated animals fully recovered to seemingly normal levels of olfactory sensitivity 45 days after the end of Mtz administration which could be indicative of DA neuron regeneration in the OB. We counted the number of CFP^+^ cells at 45 dpt and detected no differences between Mtz-treated and control animals (n = 6, *p* = 0.1352) (Fig. 6b–d). In order to demonstrate that the recovery in CFP positive cell populations was the result of newly formed cells, through a regenerative process that follows neuronal damage, we administered BrdU by intraperitoneal (i.p.) injection, starting immediately prior to Mtz treatment, recurring every 48 h, for a total of 5 i.p. injections (Fig. 6a). At 45 dpt, there was no significant difference in the total number of BrdU + cells in the OB of Mtz-treated animals compared to controls (n = 6, 925.5 vs. 941.2) (Fig. 6e–g), yet Mtz treated animals had on average 2.5-fold (student *t *test, n = 6, *p* = 0.0034) higher presence of cells positive for both BrdU and CFP in the OB when compared to controls (Fig. 6h–j). This result suggests an increase in neurogenesis of CFP^+^ cells, and thus, of DA neurons, in response to the ablation.

To further test the timing of the observed reactive neurogenesis in response to neuronal ablation, we performed a pulse-chase experiment using two different thymidine analogs, BrdU and EdU. We administered BrdU through i.p. injection as described above until day 7 post treatment for a total of 5 injections. Following BrdU administration, fish then received EdU injections at day 9, recurring every 48 h for a total of 5 injections (Fig. 6a).

At 45 dpt, there was no significant difference in the number of EdU positive (Fig. 6k–m), EdU /CFP positive (Fig. 6n–p), or BrdU/EDU/CFP- positive cells (Fig. 6q–s) in the OB of Mtz treated as compared to vehicle control animals. This suggests that there was no major increase in the production of new CFP + cells after 9 dpt in response to neuronal ablation. Overall, our data suggest, adult zebrafish are able to generate new DA neurons following ablation and that this increased production takes place within the first 9 days post ablation.

## Discussion

Here, we showed that chemogenetic ablation with the NTR-Mtz system, was efficient at ablating DA neurons within the OB of adult zebrafish. Ablation of more than half the DA neurons in the various areas of the OB was consistent with a decrease in both TH and CFP immunoreactivity, protein levels and with a reduction in dopamine levels. It also resulted in impaired olfaction in a simple repulsive stimulus test. Olfactory impairments are commonly seen in patients with PD^27–29^. Decreases in cell number, TH/CFP immunoreactivity, and olfactory performance were transient, suggesting regeneration mechanism in the adult brain. This was further supported by proliferation assays that indicated most of the new cells are formed within the first nine days after Mtz treatment.

Although neurotoxins such as MPTP and 6-OHDA have been described to induce DA neurodegeneration with PD-like pathology and phenotype in research models such as rodents, non-human primates, the planarian and salamander^30–32^, their use in the adult zebrafish did not result in consistent decreases in the numbers of brain DA neurons, despite a transient locomotor phenotype^14–17,33^. Lack of consistency in the effects of neurotoxins may be the result of difficulties in delivering the drugs to the proper brain areas. A recent study described the successful ablation of catecholaminergic clusters 5/6, 11 and 12 via intraventricular injection of 6-OHDA, yet DA clusters in the telencephalon remained unaffected^15^.

The chemogenetic approach with NTR and Mtz, which had been successful in juvenile zebrafish^20–24^ but had yet to be tested for ablation of adult, might thus be a preferred approach in DA neuron regeneration studies.

Most DA neuron losses were observed in the OB and telencephalon, following the Mtz administration regimen used in the current study (10 mM Mtz for 24 h). Despite a loss of DA neurons, a decrease in dopamine levels and impaired olfaction, we could not observe clear locomotor dysfunctions. There were no observable effects on the adult fish’s ability to perform locomotor functions such as total distance swam, swimming mechanics. Thus, it is unlikely that the inability of fish to swim away for the repulsive stimulus arm in the olfactory test could be due locomotor impairments but are rather due to the inability to detect/respond to the stimulus. It is possible that impaired olfaction occurs at a lower DA neuron loss threshold than impaired locomotion. This would be consistent with the observation that inability to smell occurs earlier to locomotor dysfunction in humans who develop PD^28,34^. Different Mtz administration regimens or the use of alternative NTR substrates such as Nifurpiridol^35^ might be necessary to achieve sufficient DA neuron losses that result in locomotor dysfunction in adult zebrafish. Finally, we cannot at this point, exclude that other functions of dopaminergic neurons such as a role in aversive behavior, could explain the phenotype observed in the olfactory test. In addition, recent work suggests that ablation of dopaminergic neurons of the substantia nigra were sufficient to impair olfaction^36^. However, the relatively larger neuronal losses in the olfactory bulb of our model remains the most likely explanation for the observed phenotype.

In contrast to mammals that show limited neurogenesis and regenerative potential in the adult brain, teleost fish have markedly higher capacity for neurogenesis and neuronal regeneration^37–39^. However, there have been only a few studies that addressed the ability of zebrafish to specifically regenerate DA neurons. Furthermore, most studies that examined the dopaminergic regeneration potential were performed in embryos/larvae^20–24^. Intracerebroventricular injections of 6-OHDA in the adult zebrafish brain were followed by DA neuronal loss in the hypothalamus, preoptic area, and posterior tuberculum (PT)^14^. Thirty days post 6-OHDA administration, DA neuron numbers had returned to values comparable to that of controls and the distribution of these neurons was also similar to that of controls. Similarly, Cadwell and colleagues showed that it took near 42 days for the re-population of DA neurons from catacholaminergic clusters 5,6,11 and 12 following 6-OHDA mediated cell ablation^15^.

Here, we showed that adult zebrafish were capable of recovering from the olfactory impairments when tested 45 days after the end of Mtz treatment. Olfactory recovery accompanied by adult neurogenesis was observed in the olfactory bulb of the mouse following 6 hydroxydopamine-mediated ablation of DA neurons^40^. Olfactory recovery was correlated with a recovery of CFP^+^ cells. In BrdU labeling experiments, the number of cells that were positive for both BrdU and CFP owere 2.5-fold higher than controls. Yet, the total number of BrdU-labeled cells were comparable between controls and Mtz-treated fish. These results suggest that most newly formed cells were DA neurons, and that zebrafish react to DA neuron ablation by stimulating the production of new DA neurons specifically, although mechanisms that regulate this process have yet to be uncovered. Pulse-chase experiments indicate that most of the newly formed CFP^+^ cells, that is, most new DA neurons were born in the first 9 days after the end of the Mtz-mediated ablation. It remains to be determined how long these newly formed DA neurons need to migrate to their final position, to integrate into circuits, and to contribute to functional recovery, such as olfaction.

PD is the second most prevalent neurodegenerative condition affecting the elderly and understanding the regenerative potential for dopaminergic neurons in the zebrafish brain may provide insights in the cellular and molecular mechanism involved in this process that may lead to novel therapeutic approaches targeting endogenous regeneration of DA neurons in PD patients.

## Conclusion

Overall, we have shown through a conditional and specific ablation approach the regenerative capacity of dopaminergic neurons in the adult zebrafish olfactory bulb. Our study shows the regenerative ability of an isolated neuron type in a non-invasive injury model. Here, neuronal regeneration led to the formation of functional neurons capable of restoring a previously described olfactory impairment. Much knowledge can be gained from studying the cellular and molecular mechanisms involved in the regenerative process described in our model with great implications for the development of therapeutic approaches aimed at neuronal injury repair such as those observed in neurodegenerative pathologies.

## Materials and methods

### Animal care and transgenic animals

All experiments were conducted in accordance with protocols approved by the University of Ottawa Animal Care Committee following guidelines of the Canadian Council on Animal Care. Homozygous Tg(*dat:CFP-NTR*)^22^ 8–12 months of age with an average body length of 3 cm were used for experiments. Fish were maintained as previously described^41^.

### Chemogenetic ablation

Adult animals were exposed to either vehicle (0.2% dimethyl sulfoxide:DMSO) (Sigma Aldrich, Oakville, Canada) or to 10 mM metronidazole (Mtz) in 0.2% DMSO dissolved in fish facility system water. Drug exposure was adapted from previously established method for the ablation of habenular neurons in the brain of adult zebrafish^42^. In summary, a maximum of 3 fish were placed into 1 L breeding tanks (Aquatic Habitats, Apopka, Florida) filled with 500 mL of either vehicle or Mtz solution. Exposure lasted 24 h and tanks were protected from light and kept in a room acclimatized to 280 °C. Upon termination of exposure, 500 mL of fresh system water were added to the exposure solution and fish were left in this tank for 30 min. Then, fish were moved into a clean 1L tank containing fresh system water and this process was repeated a total of 3 times at 1-h intervals between water exchanges. Fish were not fed during exposure. Following the wash period, fish were transferred to a 10L glass tank with an AquaClear20 power filter (Hagen, Baie d’Urfé, Canada) and feeding resumed.

### High performance liquid chromatography (HPLC)

Adult zebrafish were immobilized in ice-cold water and had their heads immediately removed. Their brains were dissected on ice cold homogenization solution (1 mM Na_2_EDTA, 0.3 M chloroacetic acid (ClCHCOOH) and 10% methanol in HPLC grade water). The olfactory bulb and telencephalic area of individual animals were removed and stored in a 2 mL microcentrifuge tube with 500 μL of homogenization solution, flash frozen on dry ice and stored at − 80 °C until further processing. DHBA was used as internal standard. Sample size was n = 6 for each time point and treatment.

Brain tissue was homogenized via sonication using a Fisher Scientific Model 100 Sonic Dismembrator (Fisher Scientific, Ottawa, Canada) and protein levels were measured using Pierce BCA protein assay kit (ThermoFisher Scientific) in a FLUOstar Galaxy system (BMG LabTech, Guelph, Canada). HPLC was carried out as previously described^22^.

### BrdU and EdU labelling

A 2.5 mg/mL stock solution of 5-bromo-2′-deoxyuridine (BrdU) (Sigma Aldrich) or 5-ethynyl-2′-deoxyuridine (EdU) (Invitrogen) was prepared in 110 mM NaCl (pH 7) fresh during the week of administration and aliquots were stored at − 80 °C protected from light for use throughout the week.

A stock solution was quickly thawed immediately prior to injections. Fish were briefly anaesthetized with MS-222 and placed in a surgical sponge designed for intraperitoneal administrations. Fish received 5  μL of BrdU solution per 0.1 g of body weight, immediately prior to exposure and repeatedly every 48 h for a total of 5 intraperitoneal (i.p.) injections. Following BrdU injections, fish received recurring EdU i.p. injections every 48 h for a total of 5 i.p. injections starting at 9 days post treatment (dpt). Fish weighed on average 0.55 g.

### Histology and immunohistochemistry

Fish were euthanized by immersion in ice-cold water followed by immediate decapitation. Heads were fixed in 2–4% methanol-free PFA (Fisher Scientific) 16 h at 4 °C. Tissue decalcification and embedding in gelatin was done as previously described^6^. Cryosections 16–25 μm thick were stored at − 20 °C until processed for immunohistochemistry. First, sections were allowed to rest at room temperature for 30 min and incubated at 37 °C for 15–30 min in PBS. Slides were washed 3 × 10 min in PBSTx (0.3% Triton-X) and heated to 80 °C in 10 mM sodium citrate for 25 min. Slides were allowed to return to room temperature in PBSTx and were incubated in primary antibody diluted in PBSTx O/N at 4 °C. For BrdU a DNA denaturing step consisting of a 20 min incubation in 2 M HCl followed by a wash in sodium borate buffer (pH 8.5) for 5 min was added prior to PBSTx washes. Following incubation in primary antibodies (Table 1), slides were washed 3 × in PBSTx at room temperature and incubated in secondary antibody for 2 h at RT and protected from light. Slides were finally washed in PBS and mounted using Vectashield mounting media (Vector Labs, Burlington, Canada).

**Table 1:** List of antibodies used and their respective dilution(s).

Host	Target	Catalog	Dilutions
Rabbit	Tyrosine hydroxylase (TH)	AB152 (Merck Millpore)	1:400
Rat	Bromodeoxyuridine (BrdU)	AB6326 (abcam)	1:400
Mouse	Cyan fluorescent protein (CFP)	632,381 (Clontech)	1:400
Goat	Rabbit IgG (H + L)	A-11012 (ThermoFisher)	1:500
Goat	Mouse IgG (H + L)	A-11001 (ThermoFisher)	1:500
Goat	Rat IgG (H + L)	A-21247 (ThermoFisher)	1:500

To detect EdU, the Click-iT™ EdU Alexa Fluor™ 647 Imaging Kit (Invitrogen) was used by following the manufacturer’s protocol. Primary antibodies used were rabbit anti-TH (Millipore, Massachusetts, USA; Cat. No. AB152), mouse anti-CFP (Clontech, Mountain View, USA; Cat. No. 632381) and rat anti-BrdU (AbCam, Cambridge, USA; Cat. No. AB6326). Secondary antibodies used were goat anti-rabbit Alexa 594, goat anti-mouse Alexa 488 and goat anti-rat Alexa 647 (ThermoFisher Scientific; Cat. No. A-11012, A-11001 and A-21247, respectively).

### Imaging and cell count

Images were obtained using either a Zeiss Axiophot epifluorescent microscope (Mercury illumination with DAPI (ex 365 nm–em 420–470 nm), GFP (ex. 450–490 nm–em 500–550 nm) and Texas Red (ex 530–585 nm–em 615 nm longpass) filter sets) or a Nikon AR1 confocal microscope (equipped with a 25 × immersion lens and with laser lines at either 405, 457, 488, 561 and 640 nm) and processed post-acquisition using Fiji software^43^. Cell counts were performed on confocal images acquired with 1 μm thick optical sections. Counts were performed by three independent researchers blinded to experimental design. Four to five sections were counted for each fish. Orthogonal views were used to assure labeling co-localization.

### Olfactory phenotype

Adult zebrafish were placed in a customized tank containing a mid-tank division which divided the tank into neutral zone, right and left arms^26^. Each animal underwent a single exposure to cadaverine. Fish were allowed to swim freely in the tank for 3 min prior to the addition of cadaverine (Sigma Aldrich) to the arm where fish was found at the 3-min time-point. The time spent in each area was recorded for another 3 min. This elapsed time was sufficient to allow the stimulus to diffuse throughout one of the tank’s arms without reaching a different tank area. The time spent in each arm and neutral zone were calculated pre and post stimulus. The ratio of time spent in stimulus arm was addressed by dividing the percent of time spent in stimulus arm post cadaverine by the percent of time spent on the same arm pre stimulus. Cadaverine (180uL) was delivered with a micropipette into the rear most portion of the arm. Experimenters were not visible to the fish and remained outside of the recording room throughout the duration of recordings except for the time when the stimulus was delivered. Control animals received the same volume of system water similarly via micropipette delivery.

### Motor phenotype

The effects of DA neuron loss on swimming parameters of adult zebrafish were addressed at 1 day before treatment or at 1 and 7 dpt with either vehicle or Mtz. Adult zebrafish exposed to Mtz and the DMSO were rinsed to remove any residual compound and placed into individual static tanks to acclimate in ambient light for 30 min prior to behavioural assessment. Trials of 10 min were performed to analyze various swimming parameters that include total swimming distance, average velocity, and freezing duration. Swimming activity was recorded using the Zebralab software and the ZebraCube tracking system (ViewPoint Life Science, Lyon, France). The tracking system consists of infrared illumination, LED lights, and a mounted camera for swimming recording under dark and light conditions. Sample sizes of n = 10 were conducted to extrapolate any chemoablative effects of Mtz apart from the natural variability in locomotion observed in adult zebrafish.

### Statistical analysis

Statistical power was calculated using the software GraphPad Prism v.8 (La Jolla, California, USA). Variability is shown as standard error from mean (SEM). Pair-wise comparison of normally distributed data was conducted via unpaired student *t *test. Zebrafish olfactory phenotype statistical power was assessed using multiple comparison *t *test with Holm–Sidak post-hock correction. Cellular loss was represented as percentage loss with reference in vehicle treated animals and 2-way ANOVA with Sidak post-hock correction was performed. A multiple *t *test comparison was used for statistical analysis of Fig. 3.

Statistical *p*-value are represented as n.s (*p* > 0.05), *(*p* ≤ 0.05), **(*p* ≤  0.01), ***(*p* ≤  0.001) and ****(*p* ≤  0.0001).
